# The Substantia Nigra Pars Reticulata Modulates Error-Based Saccadic Learning in Monkeys

**DOI:** 10.1523/ENEURO.0519-20.2021

**Published:** 2021-03-31

**Authors:** Yoshiko Kojima, Paul J. May

**Affiliations:** 1 Department of Otolaryngology- Head and Neck Surgery, Washington National Primate Research Center, University of Washington, Seattle, WA 98195-7330; 2 Department of Neurobiology and Anatomical Sciences, University of Mississippi Medical Center, Jackson, MS 39216-4505

**Keywords:** adaptation, basal ganglia, monkey, motor learning, saccade

## Abstract

The basal ganglia have long been considered crucial for associative learning, but whether they also are involved in another type of learning, error-based motor learning, is not clear. Error-based learning has been considered the province of the cerebellum. However, learning to use a robotic arm and saccade adaptation, which use error-based learning, are facilitated by motivation, which is a function of the basal ganglia. Additionally, patients with Parkinson’s disease, a basal ganglia deficit, show slower saccade adaptation than age matched controls. To further investigate whether the basal ganglia actually influence error-based learning, we reversibly inactivated the oculomotor portion of the substantia nigra pars reticulata (SNr) in two monkeys and tested saccade adaptation. Here, we show that nigral inactivation affected saccade adaptation. In particular, the inactivation facilitated the amplitude decrease adaptation of ipsiversive saccades. Consistent with previous studies, no effect was seen on the amplitude of the ipsiversive saccades when we did not induce adaptation. Therefore, the facilitated adaptation was not caused by inactivation directly modulating ipsiversive saccades. On the other hand, the kinematics of corrective saccades, which represent error processing, were changed after the inactivation. Thus, our data suggest that the oculomotor SNr assists saccade adaptation by strengthening the error signal. This effect indicates the basal ganglia influence error-based motor learning, a previously unrecognized function.

## Significance Statement

Error-based motor learning, such as learning to use a robotic arm or make accurate saccades, has been regarded as a cerebellar function. In contrast, the basal ganglia are thought to be involved in associative learning, such as associations between reward and stimulus objects, but whether they also are involved in error-based motor learning is not clear. Here, we address this question by showing that inactivation of the oculomotor basal ganglia influences the saccade motor learning, a well-established error-based motor learning model. This result suggests a previously unrecognized function of the basal ganglia.

## Introduction

The basal ganglia are thought to be involved in associative learning, such as associations between reward and stimulus objects (Doya, 2000; Shmuelof and Krakauer, 2011). In contrast, error-based learning, such as used for correcting inaccurate movements, has been regarded as a cerebellar function. However, we recently observed that the rate of saccade adaptation, a cerebellar dependent form of error-based learning (Hopp and Fuchs, 2004; Iwamoto and Kaku, 2010; Kojima, 2019; Soetedjo et al., 2019), could be facilitated by motivation (Kojima and Soetedjo, 2017a). Similarly, learning to use a robotic arm, another form of error-based learning, is also facilitated by motivation (Nikooyan and Ahmed, 2015). Since motivation is associated with basal ganglia processes underlying reward associative learning, these findings suggest that the basal ganglia may also influence error-based learning. Indeed, patients with Parkinson’s disease, which is a disorder of the basal ganglia, show slower saccade adaptation than age-matched normal controls (MacAskill et al., 2002; Abouaf et al., 2012).

Here, we examined whether the output of the basal ganglia actually influences error-based learning by using saccadic eye movements. Saccades supply a useful model for error-based motor learning for two reasons (Hopp and Fuchs, 2004; Iwamoto and Kaku, 2010; Kojima, 2019; Soetedjo et al., 2019): first, their subcortical neural circuitry is well studied (Scudder et al., 2002); and second, there is an established behavioral paradigm that causes an adaptation of saccade size by providing an apparent visual error (McLaughlin, 1967).

Previous studies have implicated the superior colliculus (SC) as the source of the error signal that drives saccade adaptation (Kojima, 2019; Soetedjo et al., 2019). First, there are disynaptic routes from the SC to the oculomotor cerebellum (oculomotor vermis; OMV). The climbing fibers that cause complex spikes in OMV Purkinje cells originate in the part of the inferior olive (IO; Yamada and Noda, 1987; Kralj-Hans et al., 2007) that receives a projection from the SC (Harting, 1977; Huerta and Harting, 1984). Second, SC stimulation can act as a surrogate error signal to drive adaptation, presumably by evoking complex spikes in the OMV (Kaku et al., 2009; Soetedjo et al., 2009). Third, elimination of the error signal by inactivating the rostral SC impairs saccade adaptation (Kojima and Soetedjo, 2018). Finally, visual activity in rostral SC neurons encodes the sensitivity of the motor error used for saccade adaptation, i.e., when this SC activity is strongest, the speed of adaptation is fastest (Kojima and Soetedjo, 2017b). All these studies taken together suggest that the SC provides an error signal to the OMV by way of the IO to drive saccade adaptation.

The substantia nigra pars reticulata (SNr) is an output station of the basal ganglia involved with the saccadic system (Hikosaka et al., 2000) that projects to and influences the SC by direct inhibition (Behan et al., 1987; Bickford and Hall, 1992). SNr neurons exhibit tonic activity during fixation, and they pause for visual stimuli and for visually-guided saccades in the contraversive direction (Hikosaka et al., 2000). During saccade initiation, there is a pause in the tonic activity of a portion of the SNr. This produces a decrease in nigrotectal inhibition allowing excitatory inputs to increase collicular activity, leading to a contraversive saccade (Hikosaka et al., 2000). Consequently, to test whether the basal ganglia can influence error-based learning, in the present study we examined the effects of inactivating the SNr when monkeys were adapting their saccades. Since inactivation of the SNr produces little direct effect on visually-guided ipsiversive saccades (Hikosaka and Wurtz, 1985), we primarily examined whether inactivating the SNr affects adaptation of ipsiversive saccades, where the results would not be confused by the direct effects of SNr inactivation on contraversive saccades. Furthermore, using this approach, we concentrated our examination on target steps that produce a motor error signal used for adaptation on the side of the SC that receives the predominant ipsilateral SNr input.

## Materials and Methods

All experiments were performed in accordance with the Guide for the Care and Use of Laboratory Animals and exceeded the minimal requirements recommended by the Institute of Laboratory Animal Resources and the Association for Assessment and Accreditation of Laboratory Animal Care International. All the procedures were evaluated and approved by the local Animal Care and Use Committee of the University.

### Surgery and training

Two male *Macaca mulatta* monkeys (E, Z) participated in this study. We implanted each monkey with fixtures to prevent head movements, a scleral search coil (Judge et al., 1980) to measure eye position in space, two recording chambers that were aimed at each side of the SNr and one recording chamber that was aimed at the SC.

After the monkeys had recovered from the surgery, we trained them to track a small visual target in a dimly lit, sound-attenuating booth. The target was a 0.3° laser spot projected onto a tangent screen via two computer-controlled orthogonal mirror galvanometers. The screen was 65 cm from the monkey’s eyes. The monkey sat in a primate chair with its head restrained. We measured eye position with the electromagnetic search coil method (Fuchs and Robinson, 1966). We rewarded the monkeys with applesauce for keeping their gaze within ±2° windows around the horizontal and vertical positions of the target spot for at least 0.5 s. Once they had learned to fixate the target spot, we trained them to make visually-guided saccades to a stepping spot that moved to random locations on the tangent screen within a ±18° radius of straight-ahead while training. We delivered the applesauce reward (∼0.16 ml per dollop, ∼200 ml/h) by a pump (Masterflex tubing pump, Cole-Parmer) every 2 s regardless of the amplitude, direction or timing of the saccade, as long as it landed within the ±2° window surrounding the target. The targeting saccade was required to occur within 0.6 s of the target step and the subsequent fixation had to be maintained for at least 0.3 s.

After the monkey reliably tracked the jumping target spot, we started recording experiments to find the region of SNr whose neurons exhibit tonic activity during fixation, and pauses for visual stimuli and for saccades in the contraversive direction (Hikosaka and Wurtz, 1983a). We used a glass-coated tungsten micro-electrode (Alpha-Omega) guided by a 21-gauge hypodermic cannula. We tested visually-guided saccades to three target amplitudes (5°, 10°, 15°) for six directions (right, right up, left up, left, left down, and right down). As in a previous study (Hikosaka and Wurtz, 1983a), neurons typically exhibited a broad response field, pausing for all three tested amplitudes with all three contraversive directions. Because of this broad response field tuning, our injection could affect a broad sector of saccade vectors.

### Muscimol injection procedure

We injected muscimol, a GABA_A_ agonist (5 μg/μl, MP Biomedicals) dissolved in a saline solution through a 36-gauge stainless steel tube. On the day preceding each injection, we made electrode penetrations to locate the SNr by recording its characteristic activity (Hikosaka and Wurtz, 1983a). On the day of the injection, we advanced the injection tube tip to 0.2 mm below the depth of the preceding electrode penetration because the tube is thicker than the electrode, and so does not penetrate as effectively as an electrode. After we collected a “preinjection” block of saccades (see Experimental procedures), we injected the muscimol by using brief pulses of air pressure (PV830 Pneumatic PicoPump, WPI).

We confirmed the location of the injection tube both physiologically and histologically. As reported previously (Hikosaka and Wurtz, 1985), spontaneous saccades contraversive to the injection side appeared about half an hour after the injection. The spontaneous saccades occurred in eight experiments (experiments #1–8; Table 1). Their occurrence most likely is caused by hyperactivity within the ipsilateral SC, which is disinhibited by SNr inactivation (Hikosaka and Wurtz, 1985). In one experiment (experiment #9; Table 1), the spontaneous saccades did not appear, suggesting that the muscimol injection failed to inactivate the appropriate region of the SNr. Probably, the tip of injection tube was not properly located in the oculomotor SNr. We analyzed this experiment as a control. We did not use saline injections as controls because saline injection sites could not be confirmed by the induction of spontaneous saccades.

**Table 1:** Summary of the conditions in all nine injection experiments

Experiment #	Monkey	SNr (Lt/Rt)	Muscimol (ul)	Adapt/noAdapt	Spontaneous saccade vector		Time to start ada (min)	
					Direction(°)	Amplitude(°)	From inj	From in booth
1	E	R	0.8	Adapt	178	5.3	19	70
2	E	R	0.6	Adapt	174	5	18	58
3	E	L	0.6	noAdapt	36	5.1	18	44
4	E	L	0.8	Adapt	16	5.5	20	100
5	Z	L	1	Adapt	17	4	17	29
6	Z	L	1	Adapt	356	2.5	17	30
7	Z	L	1	Adapt	352	3.8	17	29
8	Z	L	1.1	noAdapt	351	5	17	28
(9)	Z	(L)	0.8	Adapt	No spontaneous saccade		17	28

### Experimental procedures

**Figure 1.**
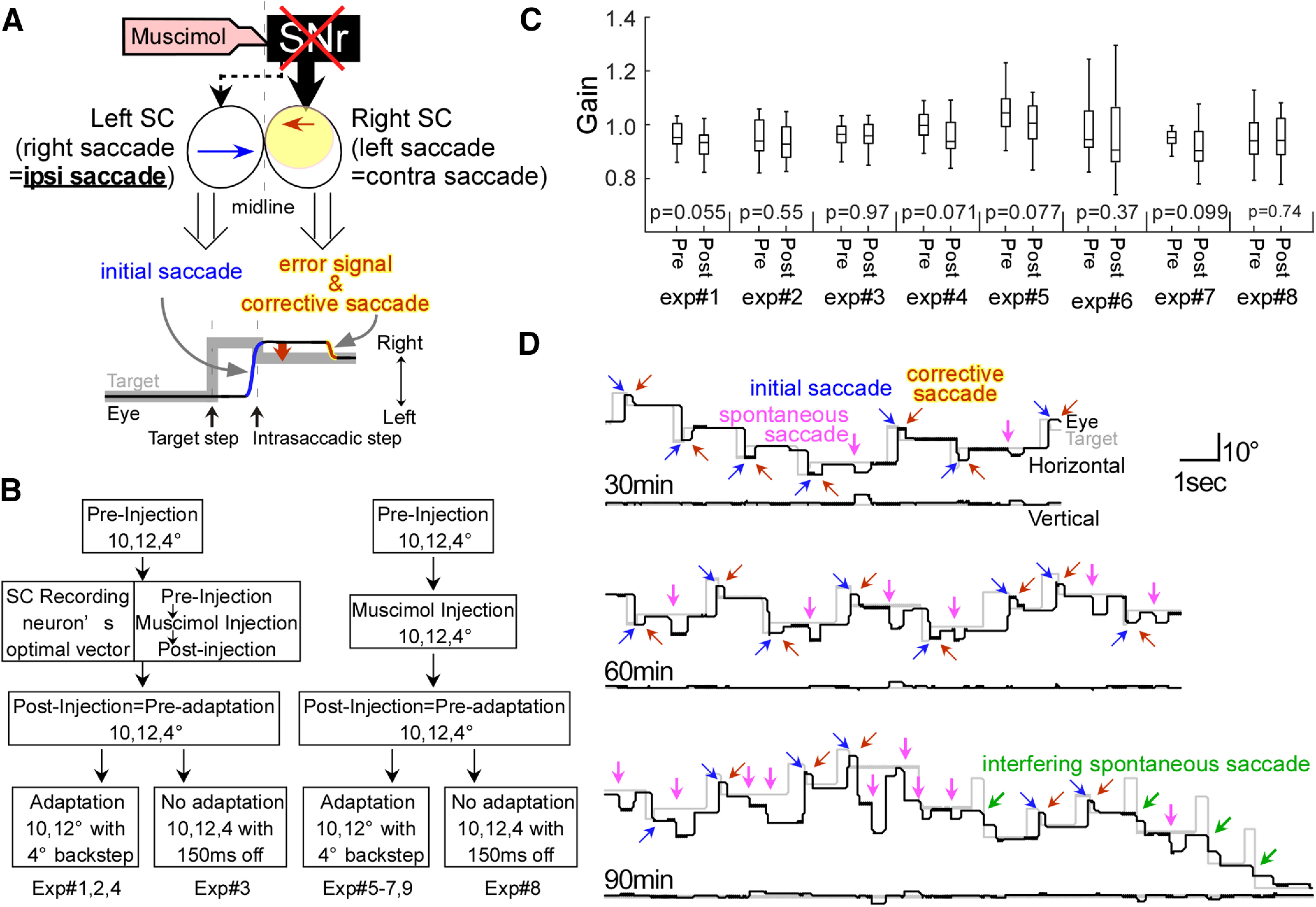
Experimental procedure. ***A***, Experimental design. The effect of SNr inactivation on ipsiversive saccade adaptation. SNr projection to ipsilateral (fat arrow) and contralateral (dashed arrow) SC. Brown arrow in the ipsilateral rostral SC represents the 4° contraversive vector that we used as the visual error to drive adaptation. Blue arrow in the contralateral caudal SC represents the ipsiversive vector (10° or 12°) of the initial saccade. ***B***, Diagram of the task. 10°, 12°, 4° are horizontal. ***C***, Effect of muscimol on the ipsiversive saccade gain. Box plots represent the first and last 25 saccades of the preinjection and postinjection period, but before adaptation session, respectively. ***D***, Development of spontaneous saccades induced by the SNr inactivation contraversive to the injection site (pink arrows) occurring at 30, 60, and 90 min after an injection into the right SNr (experiment #1). By 90 min, spontaneous saccades were quite frequent and interfered with the targeting saccades (green arrows) so we stopped the experiment. Blue and brown arrows represent adapted and corrective saccades, respectively.

In each injection experiment, we first collected at least 10 min of preinjection data (Fig. 1*B*). During this block, the monkey made visually-guided saccades to targets, which stepped by 4°, 10°, or 12° to the right or left. The three target steps occurred at random, so the starting position of each saccade was not predictable. After we had collected the preinjection data, we injected muscimol. The amount of injection for each experiment is indicated in Table 1. We collected at least 15 min of “postinjection” data. Animals continued participation in the same tasks during and after the injection.

After collecting the postinjection data, we started the adaptation. In this “adaptation” block, we caused adaptation of the 10° or 12° saccades in both directions by presenting an intrasaccadic backward target step of 4° to produce a decrease in saccade size. To keep the vector of the postsaccadic visual error constant at 4° during the entire adaptation session, we used a modified version of the McLaughlin (1967) paradigm (Robinson et al., 2003; Kojima et al., 2015; Kojima and Soetedjo, 2017b). As the monkey made a saccade toward the target, we measured the eye position at the end of the saccade (determined when eye velocity fell to 20°/s) and moved the target backward relative to that end eye position by 4°.

The muscimol injection did not affect the gain (for the definition, see below, Data analysis) of ipsiversive saccades in all eight inactivation experiments. We compared the median gains of the first 25 preinjection saccades and the last 25 postinjection saccades in each experiment (Wilcoxon rank-sum test; Fig. 1*C*). We took the last 25 postinjection saccades because the muscimol effect is more stable later than immediately after the injection (Kojima et al., 2010b). On the other hand, the injection did change the gain of contraversive saccades in three injection experiments (experiments #3, 4, 8). Because such gain changes for contraversive saccades would mask the gain change induced by adaptation, we focused on presenting adaptation data for ipsiversive saccades, although, for the sake of completeness, we present adaptation data for contraversive saccades as well.

To make sure that the muscimol itself did not affect the saccade gain, we also examined the effect of muscimol alone for the duration used during adaptation, i.e., ∼1 h (experiments #3 and 8; Table 1). Similar to the actual adaptation experiments, we collected preinjection data, injected muscimol, and then collected postinjection data. In these two experiments, instead of starting the adaptation, we continued the preinjection task (4°, 10°, or 12° to the right or left; Fig. 1*B*). In this “no-adaptation” block, we turned off the target for 150 ms after the saccades to eliminate any visual error that could cause a gain change.

Two days after an injection, the dysmetria and the spontaneous saccades had completely disappeared. We then collected behavioral control data for adaptation (“no-injection”) on each of the next 3 d. Because of the high degree of variability in adaptation from day to day (Hopp and Fuchs, 2004), we collected three no-injection experiments for each injection experiment to make sure the difference between no-injection and injection experiments was not because of the adaption variability. We started the no-injection adaptation trials (for experiments #1, 2, 4, 5, 6, 7, 9) or no-injection no-adaptation trails (for experiments #3, 8) after the monkey had made about the same number of preinjection, during injection, and postinjection trials for the same amount of time as in an injection experiment. The adaptation was induced using the same saccade vectors as in the injection experiment.

### Data analysis

We digitized eye and target position signals at 1 kHz and sampled unit activity at 50 kHz using Power 1401 data acquisition/controller hardware (Cambridge Electronic Design). We saved data to a hard disk for later analysis. During the experiment, Spike2 (Cambridge Electronic Design) controlled target movement and the monkey’s reward via the Power 1401 hardware.

We used Spike2 to analyze the saved data. It detected saccades when eye velocity exceeded 75°/s within 50–800 ms after a target jump. The program marked saccade onset and end when the eye velocity vector exceeded or fell below a 20°/s threshold, respectively. The program measured several saccade attributes including amplitude, peak velocity and duration, as well as the distance to the target at the beginning of each saccade. We exported saccade attributes and target positions to MATLAB (MathWorks) to analyze their relationships. We eliminated saccades whose initial eye positions differed from those of the initial target positions by >5°.

The total number of trials during adaptation differed from experiment to experiment. All datasets contained at least 472 ipsiversive saccade trials during adaptation; therefore, we analyzed only the first 472 trials in all injection and no-injection experiments. For contraversive saccade, we analyzed 373 trials.

To document the amplitude of the initial saccade to be adapted (10° or 12° target steps), we calculated the vector gain of each saccade. To normalize trial-to-trial differences of initial eye position before a saccade, the target step size was computed relative to the initial eye position:

$$Vector\;Gain(n)=\frac{\sqrt{{\left(HEi(n)-HEe(n)\right)}^{2}+{\left(VEi(n)-VEe(n)\right)}^{2}}}{\sqrt{{\left(HEi(n)-HTe(n)\right)}^{2}+{\left(VEi(n)-VTe(n)\right)}^{2}}},$$

where (*n*) is *n*^th^ trial, HEi is horizontal initial eye position, HEe is horizontal eye end position, VEi is vertical initial eye position, VEe is vertical eye end position, HTe is horizontal target end position, and VTe is vertical target end position.

To document the adaptation, the vector gain was normalized by the median of vector gain of the initial 25 adaptation trials of the experiment:

Gain(n)=1−(Median vector gain(1→25)–Vector Gain(n)).

We calculated the gain of corrective saccades in the same way.

To compare the progression of adaptation between the injection and no-injection experiments, we fitted the course of each adaptation with an exponential function (Straube et al., 1997; Kojima and Soetedjo, 2018):

$$f_{exponential}\left(n\right)=a\times exp(-b\times n)+c.$$

We tested whether an injection experiment and a no-injection experiment produced different exponential fits by means of an overall *F* test for regression (Motulsky and Christopoulos, 2004). Briefly, in the null hypothesis one exponential fits all the data points from both the injection and no-injection datasets. The alternative hypothesis is that the fits are different. We calculated the *F* ratio.

F − ratio=((SSnull−SSalt)/SSalt)/((DFnull−DFalt)/DFalt),

where SSnull is the percent difference of the sum-of-squares of errors from the null hypothesis fit. SSalt is the percent difference of the sum of the two sums-of-squares of errors from the two alternative hypotheses fits, i.e., injection data versus combined data points (injection and no-injection control) and no-injection control versus combined data points. DFnull and DFalt are the percent difference in their degrees of freedom, respectively. If the null hypothesis is true, the *F* ratio is 1.0. We then computed a *p* value from the *F* distribution. When *p* < 0.05, we consider that the data from the injection and each no-injection experiment were significantly different.

We also calculated the amount of gain change in the injection and each no-injection experiment:

(1) $$Amount\;of\;gain\;change=f_{exponential}\left(472\right)–f_{exponential}.$$

To evaluate the injection effect on the change in saccade parameters, that is, gain, reaction time, and peak velocity, we used a mixed-effect model (MATLAB function *fitlme*; Biostatistics Consulting service of the University; Kleinbaum et al., 1987). For adaptation experiments, we combined all six datasets (six injections and eighteen no-injections) and fitted with a linear mixed-effect model:

$$log\left(saccade\;parameter\right)=\;b_{0}\;+\;\;b_{1}injection\;+\;\;b_{2}trial+\;b_{3}\;injection\times \;trial\;+\;random\;effect\;monkey+\;random\;effect\;dataset\;+\;random\;effect\;condition.$$

For no-adaptation experiments, we combined both datasets (two injections and six no-injections for no-adaption datasets) and fitted with a linear mixed-effect model:

$$log\left(saccade\;parameter\right)=b_{0}\;+\;b_{1}injection\;+\;b_{2}trial+\;b_{3}injection\times trial\;+\;random\;effect\;monkey+random\;effect\;condition,$$

where *injection* is 1, if the muscimol was injected, and 0, if it was a no-injection control experiment. The *random effect condition* is “injection” or “no-injection.” We examined the coefficient of the injection × trial variable and determined the exponential of the value, that is, e^b3^. This was interpreted as the ratio of time trends comparing an injected experiment to a no-injection experiment. For example, suppose the value of e^b3^^(472–1) was 0.96 for the gain of the adaptation experiments. This can be interpreted as: “gain change for the injection experiments for 472 trials was 4% faster than in the no-injection experiments.”

To display the course of change during adaptation in the saccades across all datasets, we computed the trial population average (MATLAB function *mean*). We calculated the logarithm of the reaction times and peak velocity because their distribution usually was not Gaussian, but had a long tail.

$$\begin{array}{l} Trial\;population\;average(n)\\ =m^{-1}\sum_{m=1}^{m}\left(saccadeparameter(n)\right) \end{array}$$

where *m* is number of experiments (6 for adaptation injection experiments, 18 for adaptation no-injection, 2 for no-adaptation injection, 6 for no-adaptation no-injection). To smooth the course of the change, we calculated the moving average over a sliding window of 50 trials (MATLAB function *movmean*).

Population average = moving average (trial population average)

In all the statistical analyses, when *p* < 0.05, we considered the data from the injection and no-injection experiments to be significantly different.

### SC recording

In one monkey (E; experiments #1–4), we recorded the rostral SC activity before and after the SNr inactivation. Once we collected a preinjection block of saccades (Fig. 1*B*), we drove an electrode into the intermediate layer of the SC and isolated neurons. We recorded from SC neurons ipsilateral to the SNr injection site because most of the SNr projection to the SC is ipsilateral (Jayaraman et al., 1977; Beckstead et al., 1981; Hikosaka and Wurtz, 1983b). We isolated a SC neuron while the monkey made saccades to target steps with pseudorandom vectors. To identify the neuron’s optimal vector, we first examined the burst associated with target steps and saccades angled every 10°, and then determined the angle halfway between target step directions where the burst was the weakest (Sparks and Mays, 1980; Munoz and Wurtz, 1995). In the optimal direction, we then examined the burst for amplitudes that varied in 0.5° increments and determined the optimal amplitude with the strongest burst (Soetedjo et al., 2002a,b; Kojima and Soetedjo, 2017b). Because stimulation of the rostral SC induces greater adaptation than stimulation of the caudal SC (Kaku et al., 2009; Soetedjo et al., 2009), we focused on the rostral SC, whose neurons respond best to small target and/or saccade amplitudes. Once we had identified the neuron’s optimal vector, we collected activity for visually-guided saccades along the optimal vector (Fig. 1*B*, preinjection next to SC recording). Once we had collected the preinjection data, we drove the pipette to the SNr and injected while maintaining the isolation of the SC neuron. We then collected the SC activity for the same saccades that we collected before the injection. In 2 experiments (experiments #1, 3), we lost isolation of the unit, so we discontinued the recording and started the next block (Fig. 1*B*, postinjection = preadaptation 10°, 12°, 4°).

We digitized the sampled unit activity at 50 kHz using Power1401 data acquisition/controller hardware (CambridgeElectronicDesign). A voltage threshold detected each action potential and the program saved its time of occurrence. The marked events were carefully checked by eye (Kojima and Soetedjo, 2017b).

### Neuroanatomy

In one monkey (E), we injected biotinylated dextran amine (BDA; 10,000 MW, Invitrogen, Thermo Fisher Scientific) into the right rostral SC through a 35-gauge stainless steel tube. The tube was insulated by epoxylite except for its beveled tip to allow electrical stimulation. To prepare for the injection, we plotted the topographic map of the rostral SC (Robinson, 1972; Sparks and Mays, 1980; Munoz and Wurtz, 1995) by recording unit activity and using electrical stimulation. On the day preceding each injection, we made electrode penetrations into the SC to reveal the optimal vector of that locus (Sparks and Mays, 1980; Munoz and Wurtz, 1995; Soetedjo et al., 2002a,b; Kojima and Soetedjo, 2017b) by recording and stimulation (50 μA, 500 Hz, 50-ms trains of 0.1-ms cathodal pulses). On the day of the injection, we advanced the tip of the injectrode until we heard neuronal bursts related to pseudo-random (in direction and size) target steps and/or the targeting saccades (Kojima and Soetedjo, 2018). We then stimulated to evoke saccades and took the site’s preferred vector as the average vector of five evoked saccades. At a site where stimulation evoked a 1.7° saccade in a 144° direction (left and up), we injected 120 nl of 10% BDA by using brief pulses of air pressure (PV830 Pneumatic PicoPump, WPI).

Twenty-two days after the BDA injection, the animal was perfused with 4% paraformaldehyde in 0.1 m, pH 7.2 phosphate buffer (PB). Serial sections of the brain were cut in the frontal plane using a Vibratome (Leica) at a thickness of 100 μm. To visualize the BDA, sections were first incubated in avidin conjugated to horseradish peroxidase diluted 1:500 in PB with 0.03% Triton X-100 for 24 h at 4°C. They were then reacted with the chromagen, diaminobenzidine HCl (DAB). This process consisted of an incubation in 0.5% DAB in 0.1 m, pH 7.2 PB, with 0.005% cobalt chloride and 0.01% nickel ammonium sulfate. The reaction was catalyzed by the addition of 0.005% hydrogen peroxide and stopped by rinses in PB. Sections were then mounted onto gelatinized slides, counterstained with cresyl violet, dehydrated, cleared and coverslipped. Alternatively, they were counterstained using the cytochrome oxidase method (Wong-Riley, 1989). Images of the labeling were taken with a Nikon Eclipse E600 microscope equipped with a DS-Ri1 digital camera. Nikon Elements software controlled the image acquisition and Adobe Photoshop was used to adjust the color and contrast of the images to more closely match the appearance of the material to the eye.

## Results

We tested the consequences of unilateral muscimol injections into the SNr. Figure 1*A* illustrates our experimental design. Because most of the SNr projection to the SC is ipsilateral (Jayaraman et al., 1977; Beckstead et al., 1981; Hikosaka and Wurtz, 1983b), SNr inactivation mainly affects the ipsiversive SC, which encodes error signals and corrective saccades during the adaptation paradigm (right SC; leftward saccade, brown arrow). The other side of the SC, which, in our experiment, encodes the initial saccades (left SC, rightward saccade, blue arrow), is less affected by the injection because of the much smaller crossed nigrotectal pathway. As indicated in Figure 1*A*, bottom, an initial saccade is made to the location of the initial target. When the target is moved decreasing the target distance by 4°, the result is a target error signal that results in a second, corrective saccade. During adaptation, the gain of the initial saccade is decreased, so we will refer to these eye movements as adapting saccades in the remaining text.

Table 1 shows the details of each experiment, including the participating monkey, the side of the injection, the amount of drug injected, the behavioral paradigm employed, and the time between the injection and (1) the start of the adaptation session and (2) when the monkey started working in the booth. Each muscimol injection experiment consisted of four blocks: preinjection, during injection, postinjection, and adaptation or no-adaptation (Fig. 1*B*). While the preinjection, during injection, postinjection, and no-adaptation blocks were taking place, the monkey made visually-guided saccades to target steps of 4°, 10°, or 12° to the right and left. During the adaptation block, we induced a gain decrease adaptation of 10° and 12° saccades to the right and left by creating a 4° visual error with a backward intrasaccadic target step. In seven experiments, we examined saccade adaptation after a muscimol injection (Fig. 1*B*; Table 1, experiments #1, 2, 4–7, 9). In two control experiments, we examined the long-term effect of muscimol on saccades that did not undergo adaptation (experiments #3, 8). In four experiments (experiments #1–4), we recorded rostral SC activity before and after the SNr inactivation. Each injection experiment was followed on subsequent days by three no-injection control experiments. Therefore, one dataset consists of four experiments, that is, one injection and three no-injection experiments.

In agreement with previous reports (Hikosaka and Wurtz, 1985), muscimol injections into the SNr in this study produced little effect on the gain of ipsiversive saccades (Fig. 1*C*; Wilcoxon rank-sum test, *p* > 0.05 in all eight inactivation experiments).

We confirmed the injection site behaviorally and histologically. As reported previously (Hikosaka and Wurtz, 1985), spontaneous saccades contraversive to the injection side appeared during fixation about a half hour after the SNr injection (Fig. 1*D*, 30 min, pink arrows). This is because inactivation of the SNr disinhibits the ipsilateral SC, which provides the saccade commands for contraversive saccades (Hikosaka and Wurtz, 1985; Fig. 1*A*). We considered the appearance of these spontaneous saccades as an indication that the muscimol injection had successfully inactivated the appropriate region of the SNr. The spontaneous saccades occurred in eight experiments (experiments #1–8; Table 1). Despite the appearance of spontaneous saccades that gradually increased in frequency after the injection, monkeys still could make targeting saccades and corrective saccades (Fig. 1*D*, 60 min). Therefore, adaptation could be induced. However, by 90 min, spontaneous contraversive saccades became so frequent that they interfered with the targeting saccades (Fig. 1*D*, 90 min, green arrows). At that point, adaptation became impractical, and we stopped the experiment. The average vector of the spontaneous saccade during the adaptation session for each experiment is indicated in Table 1. The amplitude of spontaneous saccades increased gradually (1.1°/30 min on average). The average amplitude of the spontaneous saccades in all eight experiments was 4.5°, suggesting that our injection inactivated the ∼4° area of the SNr.

In one experiment, we injected muscimol and examined saccade adaptation, but the spontaneous saccades did not appear, indicating that the injection failed to inactivate the oculomotor SNr (experiment #9). We analyzed this experiment as an additional control.

**Figure 2.**
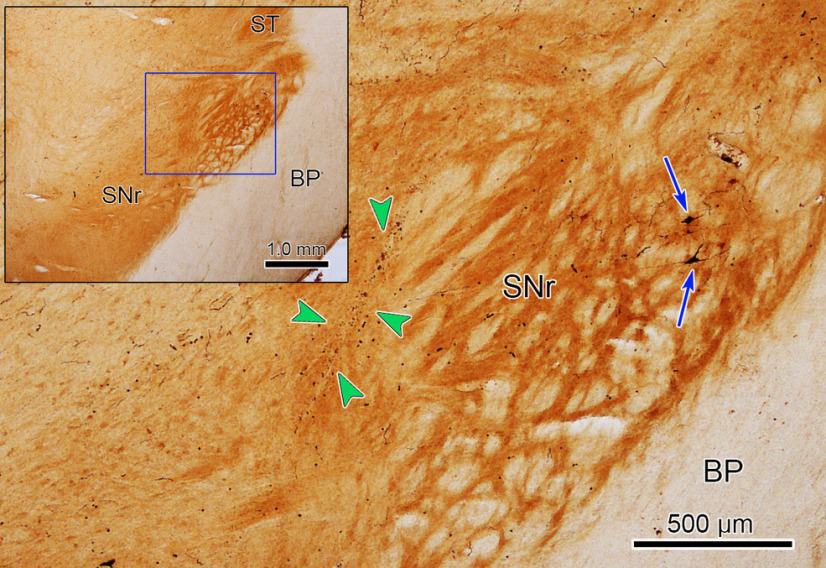
Histologic confirmation of the injection area (Monkey E). An example of an image from a cytochrome oxidase-stained section containing the substantia nigra is shown in the inset, upper left. The boxed area is shown at higher magnification in the rest of the figure. Several nigrotectal neurons that were retrogradely labeled from a BDA injection in the rostral SC are indicated by blue arrows. An injectrode track (green arrowheads) is located adjacent to this population. BP, basis pedunculus; SNr, substantia nigra pars reticulata; ST, subthalamic nucleus.

In one animal, we confirmed the injection site histologically. Figure 2 shows the muscimol injection pipette track (green arrow heads) in the SNr. In addition, to confirm that the injection area in the SNr was the eye movement region that projects to the rostral SC, we injected BDA at a physiologically specified location (see Materials and Methods) in the rostral SC of Monkey E. Figure 2 shows BDA labeled SNr neurons (blue arrows) near the injection pipet track (green arrow heads) indicating the muscimol was delivered near the appropriate nigrotectal neurons that affect small amplitude saccades.

### Effect of muscimol injections on saccade adaptation

**Figure 3.**
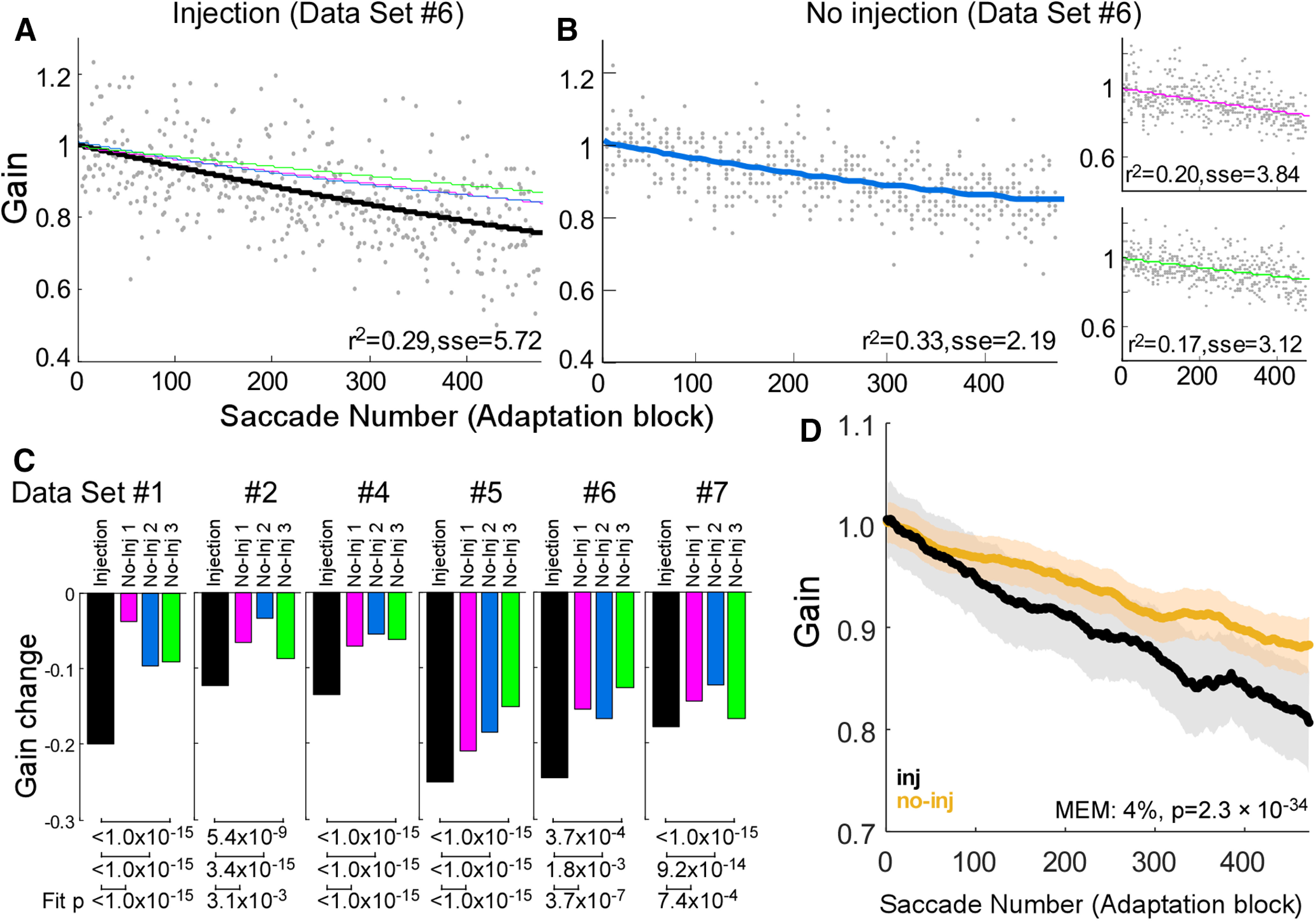
Gain change during adaptation. ***A***, Time course of the adaptation in a representative injection experiment (dataset #6). Gray dots represent individual adapting saccades and a black line is the exponential fit for the data. Three colored lines are exponential fits for the three associated no-injection control experiments shown in ***B***. (Note that the pink and blue lines are overlapped.) Goodness of fit (*r*^2^ and sse) is indicated at bottom of the plot. ***B***, Three associated no-injection control experiments (dataset #6). Gray dots represent individual saccades and colored lines are exponential fits to the data. ***C***, Amount of gain change for all six datasets. Black bars indicate the amount of gain change in injection experiments; colored bars indicate the three no-injection control experiments associated with each injection; *p* values at bottom of bar graph indicate that the exponential fits for the injection and associated control datasets are significantly different in all datasets. ***D***, Population average of adapting saccades across all datasets (six injection experiments and 18 no-injection experiments). Black line is injection experiments and light brown line is no-injection experiments. The tinted shadows indicate SEM for injection and no-injection experiments, respectively. Result from the mixed effect model (MEM) is indicated at bottom of the plot.

Figure 3*A* shows the effect of a SNr inactivation on the course of the gain change for a representative dataset during adaptation (experiment #6). The exponential fits of the course of adaptation after the injection (Fig. 3*A*, black line) showed a faster gain decrease than did the no-injection adaptations (Fig. 3*A*,*B*, blue line). The total gain change was larger in the injection than no-injection experiments in all six datasets (Fig. 3*C*). For all datasets, the fits for the injection experiment were significantly different from any of the three associated no-injection experiments (Fig. 3*C*, under bar graph; *F* test, *p* < 0.05).

To display the course of gain change across all six datasets (six injection experiments and 18 no-injection experiments), we computed the population average (Fig. 3*D*). The average gain change of the injection experiments was greater than that of the no-injection experiments. For group statistics, we used a mixed-effect model to test this effect (Table 2). It indicated that the gain change in injection experiments was 4% faster than that in the no-injection experiments (mixed-effect model b3, *p* =2.3 × 10^−34^). Thus, inactivation of the SNr facilitated saccade adaptation.

**Table 2.** Summary of the mixed effect model findings

		Mixed-effect model		%	*p*	%	*p*	%	*p*
Ipsi	Adapt	Injection	b1	−64.32	0.808	−3.42E+25	1.1E-35	−7.76E + 09	0.119
		Trial	b2	6.29	0.0E+00	−3.01	1.8E-21	4.16	9.9E-22
		Injectiontrial	b3	3.89	2.3E-34	−3.18	4.9E-07	2.97	6.7E-04
	No adapt	Injection	b1	100.00	3.6E-04	−4.76E+15	1.2E-29	96.9	0.546
		Trial	b2	0.73	4.1E-03	−2.48	1.3E-06	−4.32	7.6E-07
		Injectiontrial	b3	0.76	0.138	1.24	0.217	−4.48	1.0E-02
Contra	Adapt	Injection	b1	82.29	0.369	91.46	0.621	−5.44E+19	4.2E-16
		Trial	b2	3.41	7.7E-77	−2.07	3.0E-06	1.43	8.4E-03
		Injectiontrial	b3	−2.79	1.1E-13	10.43	9.6E-36	−4.70	2.4E-05
	No adapt	Injection	b1	−1.26E + 03	0.244	−1.36E+13	5.8E-05	91.1	0.629
		Trial	b2	1.67	2.9E-12	−2.64	1.5E-05	4.33	5.2E-08
		Injectiontrial	b3	−0.94	0.052	9.19	1.5E-15	−9.37	3.6E-08

### No-adaptation control experiments

**Figure 4.**
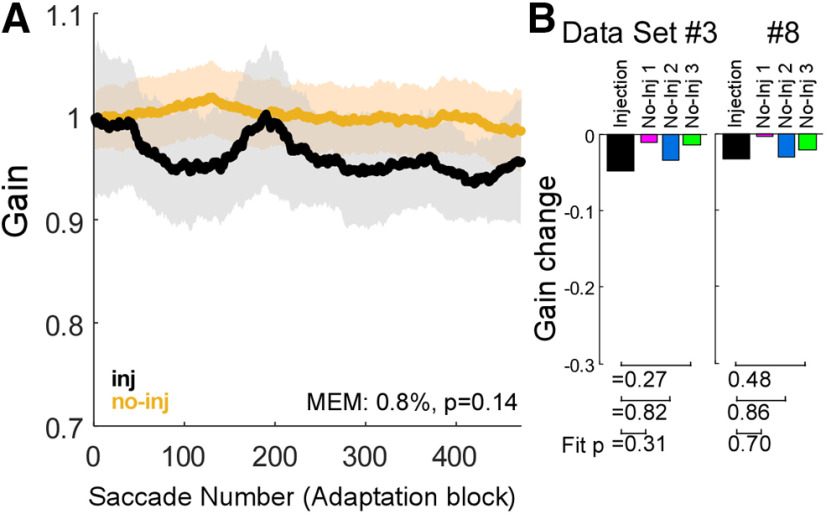
Gain change of 10° and 12° saccades during no-adaptation control experiments. ***A***, Population average of both datasets (experiments #3, 8: two injection experiments and six no-injection experiments). Same organization as in Figure 3*D*. ***B***, Amount of gain change. Same organization as in Figure 3*C*.

To rule out the possibility that the muscimol might gradually affect ipsiversive saccade gain for the many saccades required for adaptation, we examined saccade gain without adaptation in two control experiments (experiments #3, 8; Table 1; Fig. 1*B*). Figure 4*A* shows the population average of the gain change during the no-adaptation block across two datasets (two injection experiments and six no-injection experiments). The group test indicated that the gain change of injection experiments was only 0.8% greater than no-injection experiments, and was not significantly different from controls (mixed-effect model b3, *p* = 0.14). Furthermore, the group test indicated that the gain of injection experiment was nearly 100% lower than no-injection experiments when they are in the same trial (mixed-effect model b1, *p* = 3.6 × 10^−4^). The trial-by-trial gain change in trials with the same injection status was only 0.7% (mixed-effect model b2, *p* =4.1 × 10^−3^). In addition, the exponential fits for the injection experiment were not significantly different from any of the three associated no-injection experiments (Fig. 4*B*, under bar graph of amount of gain change; *F* test, *p* > 0.05). Thus, inactivation of the SNr significantly altered the gain, but did not significantly alter the gain change during no-adaptation sessions.

### Failed inactivation control dataset

**Figure 5.**
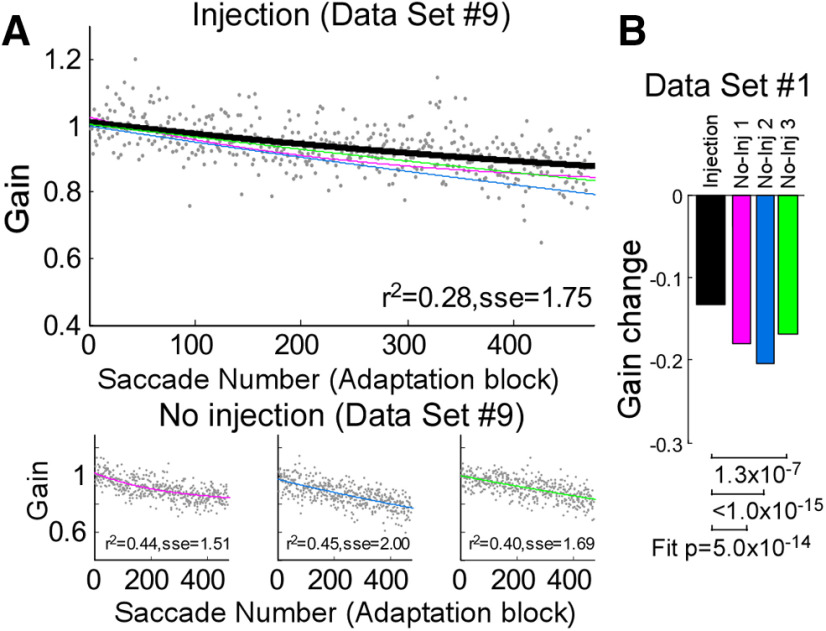
Gain change during adaptation in the failed inactivation experiment. Same organization as in Figure 3*A–C*. ***A***, Gain change in an injection experiment (top) and no injection experiments (bottom). ***B***, Amount of gain change.

Figure 5 shows the gain change during the one experiment in which the muscimol injection did not produce spontaneous saccades, i.e., failed to inactivate the oculomotor portion of the SNr. In contrast to the successful injections (Fig. 3), the adaptation after this injection (black line) showed a slower gain decrease than the no-injection adaptations (Fig. 5*A*, pink, blue, and green lines). In addition, the amount of gain change was smaller in the injection than in the no-injection experiments (Fig. 5*B*). The exponential fits for this injection experiment were significantly different from any of the three associated no-injection experiments (Fig. 5*B*, statistics beneath bar graph; *F* test; see Materials and Methods). A group test (mixed-effect model) also indicated that the gain change of injection experiments was 3% slower than in the no-injection experiments (*p* = 7.6 × 10^−7^). Thus, adaptation was not facilitated when the pipette was inserted into a location where the oculomotor output of the SNr was not inactivated.

### Changes in reaction time and peak velocity during adaptation

**Figure 6.**
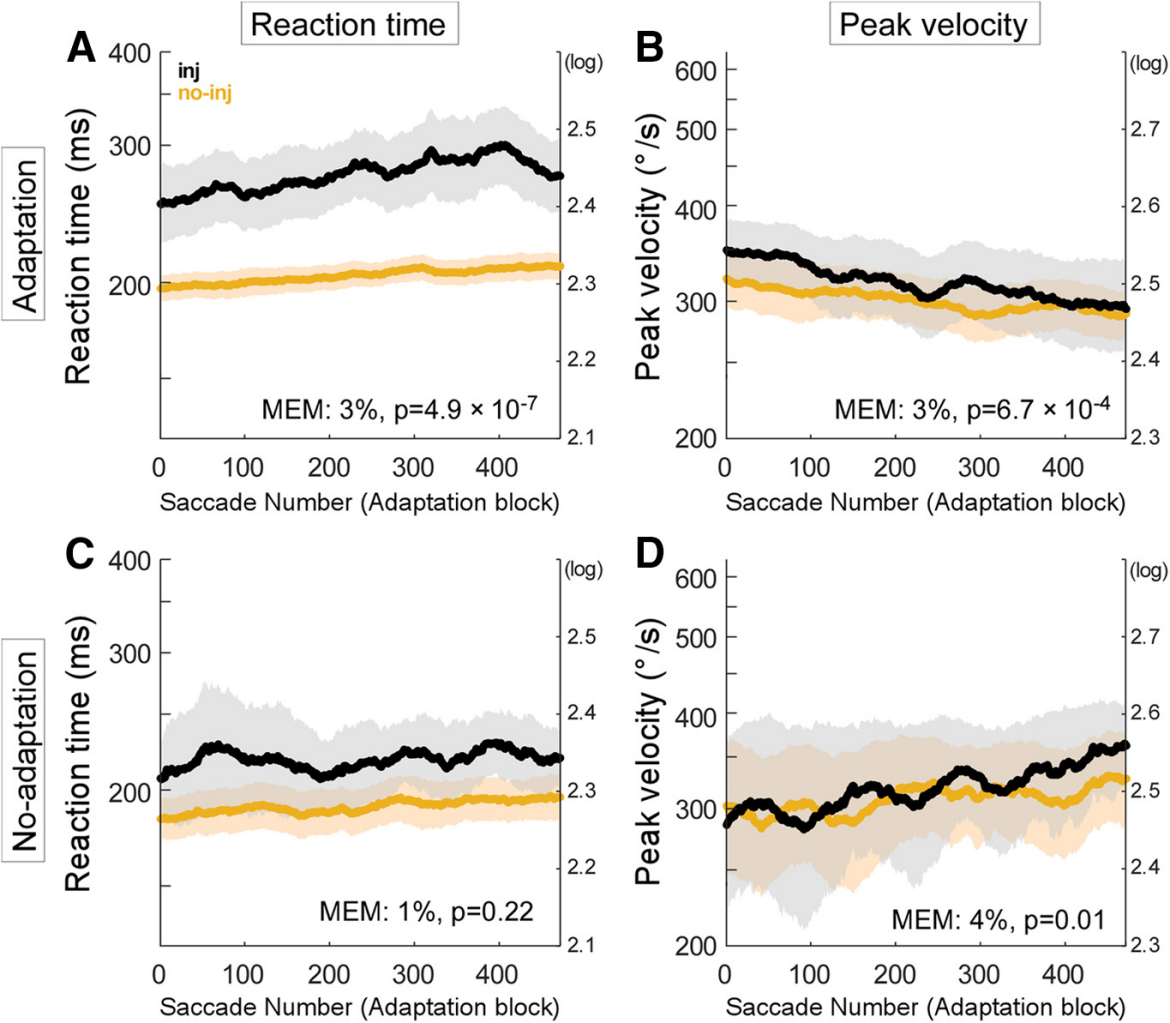
Reaction time and peak velocity of adapting saccades. Same organization as in Figure 3*D*. ***A***, Reaction time of adaptation datasets. ***B***, Peak velocity of adaptation datasets. ***C***, Reaction time of no-adaptation datasets. ***D***, Peak velocity of no-adaptation datasets.

Figure 6 examines possible changes in the reaction time and peak velocity of adapting saccades. Each plot displays the population average of all 6 datasets (experiments #1, 2, 4–7; Table 1). The saccadic reaction time in injection experiments was longer at the beginning and increased faster during adaptation than in no-injection experiments (Fig. 6*A*). The mixed-effect model test of all six datasets indicated that the increase of reaction time in injection experiments was 3% faster than in no-injection experiments (*p* = 4.9 × 10^−7^). The velocity also changed. Peak saccadic velocity in injection experiments was slightly higher at the beginning and decreased faster during the adaptation than did the velocity of the no-injection experiments (Fig. 6*B*). Note that the effect of this decrease was that there was no difference between the peak velocity of the injection and no-injection data at the end of the trials. The mixed-effect model of all six datasets indicated that the decrease in the peak velocity in injection experiments was 3% faster than the decrease in the no-injection experiments (*p* = 6.7 × 10^−4^).

Figure 6*C*,*D* shows the population average of the no-adaptation datasets (experiments #3, 8; Table 1). The reaction time in the injection experiments was longer at the beginning than in the no-injection experiments. Both the injection and no-injection reaction times increased slightly over the trials, but their increases were not significantly different from each other (Fig. 6*C*, mixed-effect model, *p* = 0.22). The population averages for peak velocity showed similar small increases over the trials in both the injection and no-injection experiments (Fig. 6*D*), and only slightly diverged in the last 100 trials. Although it is not obvious in the plot of the population averages, the mixed effect model indicated that the peak velocity for the injection experiments increased 4% more than for the no injection experiments (*p* = 0.01). This apparent paradox is because the mixed-effect model considers individual factors, such as dataset and monkey, while the plot of population average does not.

**Figure 7.**
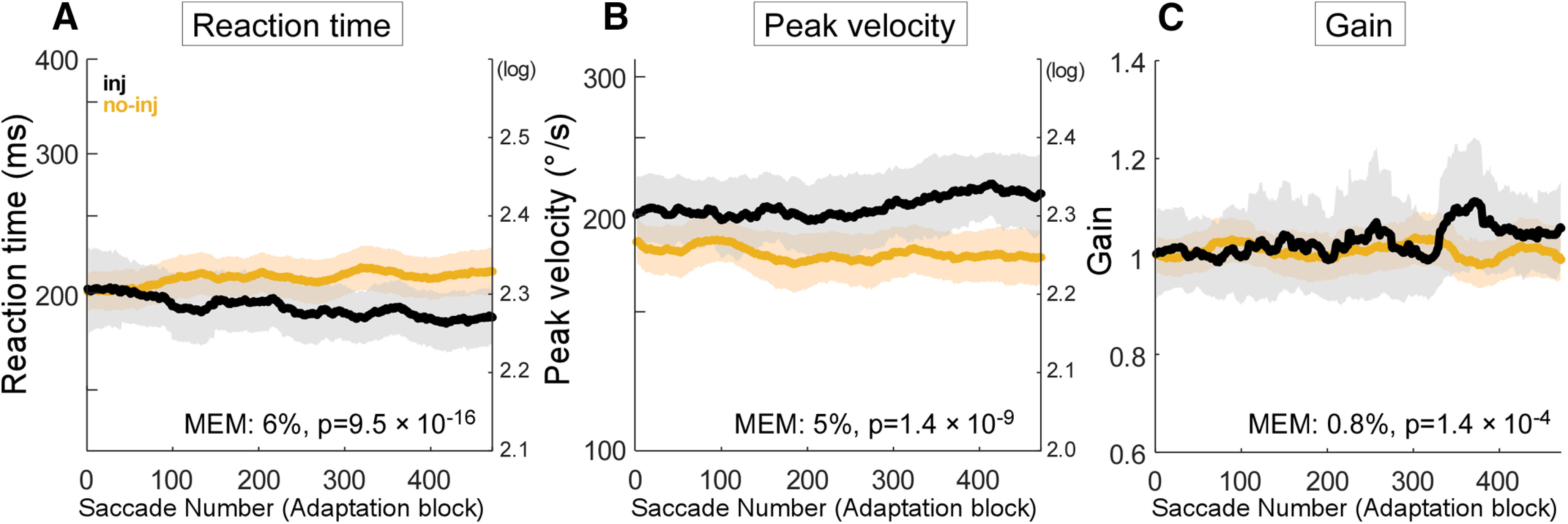
Corrective saccades during adaptation. Same organization as in Figure 3*D*. ***A***, Reaction time of corrective saccades in adaptation datasets. ***B***, Peak velocity of corrective saccades in adaptation datasets. ***C***, Gain of corrective saccades in adaptation datasets.

We also analyzed changes in the reaction time and peak velocity of corrective saccades during adaptation. Figure 7 displays the population average of these parameters in all six adaptation datasets (experiments #1, 2, 4–7; Table 1). Note that there are no corrective saccades in the no-adaptation datasets (experiments #3, 8; Table 1). The reaction times of corrective saccades during adaptation decreased in injection experiments, but they did not change during no-injection experiments (Fig. 7*A*). The mixed-effect model indicated that the decrease in the reaction times in injection experiments was 6% greater than that of the unchanging no-injection experiments (*p* = 9.5 × 10^−16^). The peak corrective saccade velocity in injection experiments was higher at the beginning and increased somewhat during the last 200 adaptation trials, whereas in the no-injection experiments, it decreased slightly over the trials (Fig. 7*B*). The mixed-effect model indicated that the peak velocity in the injection experiments was 5% greater than in the no-injection experiments (*p* = 1.4 × 10^−9^).

Because we kept the visual error constant at 4° during adaptation in all experiments by stepping from the saccade end, instead of the initial target (Materials and Methods), we expected that the corrective saccade amplitude would not change during adaptation, and that there would be no difference between injection and no-injection experiments. To confirm this expectation, we computed the population average of corrective saccade gain (Fig. 7*C*). It increased only slightly during the last 100 trials in the injection experiments. The mixed effect model indicated the increase was only 0.8% greater than that of the no-injection experiments (*p* = 1.4 × 10^−4^).

### Contraversive saccades

**Figure 8.**
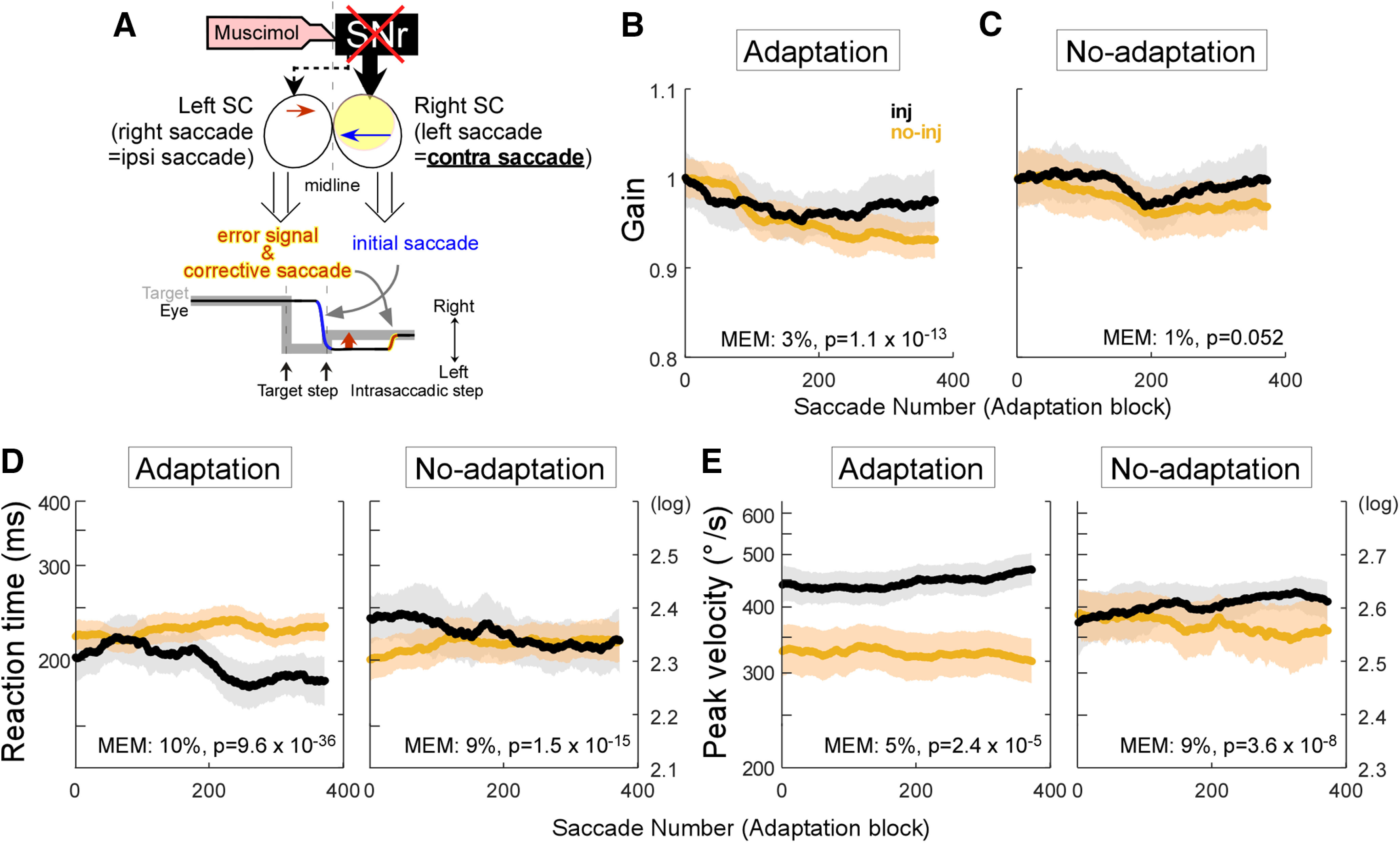
Contraversive saccade effects. ***A***, The effect of SNr inactivation on contraversive saccades. Same organization as in Figure 1*A*. ***B–E***, Population average. Same organization as in Figure 3*D*. ***B***, Gain of adaptation datasets. ***C***, Gain of no-adaptation datasets. ***D***, Reaction time of adaptation (right) and no-adaptation (left) datasets. ***E***, Peak velocity of adaptation (right) and no-adaptation (left) datasets.

Figure 8*A* illustrates the effect of SNr inactivation on contraversive saccades where 10° and 12° saccades are made to the left and the target was shifted to the right to induce adaptation. The SNr inactivation mainly affects the right SC, which encodes the initial leftward saccades (blue arrow). Figure 8*B* shows the population average of the gain change during the adaptation across 6 adaptation datasets. The adaptation of the injection experiments was slower than that of the no-injection experiments (mixed-effect model, 3%, *p* = 1.1 × 10^−13^). In the no-adaptation datasets, the gain change was not significantly different in injection and no-injection experiments (Fig. 8*C*, mixed-effect model, *p* = 0.052). Saccadic reaction time decreased in the injection experiments in both adaptation (mixed-effect model, 10%, *p* = 9.6 × 10^−36^) and no-adaptation (mixed-effect model, 9%, *p* = 1.5 × 10^−15^) trials, while it slightly increased in no-injection experiments (Fig. 8*D*). Peak saccadic velocity was somewhat variable, but it increased in the injection experiments in both adaptation (mixed-effect model, 5%, *p* = 2.4 × 10^−5^) and no-adaptation (mixed-effect model, 9%, *p* = 3.6 × 10^−8^) trials, while it slightly decreased in the no-injection experiments (Fig. 8*E*).

### SC activity before and after the SNr inactivation

**Figure 9.**
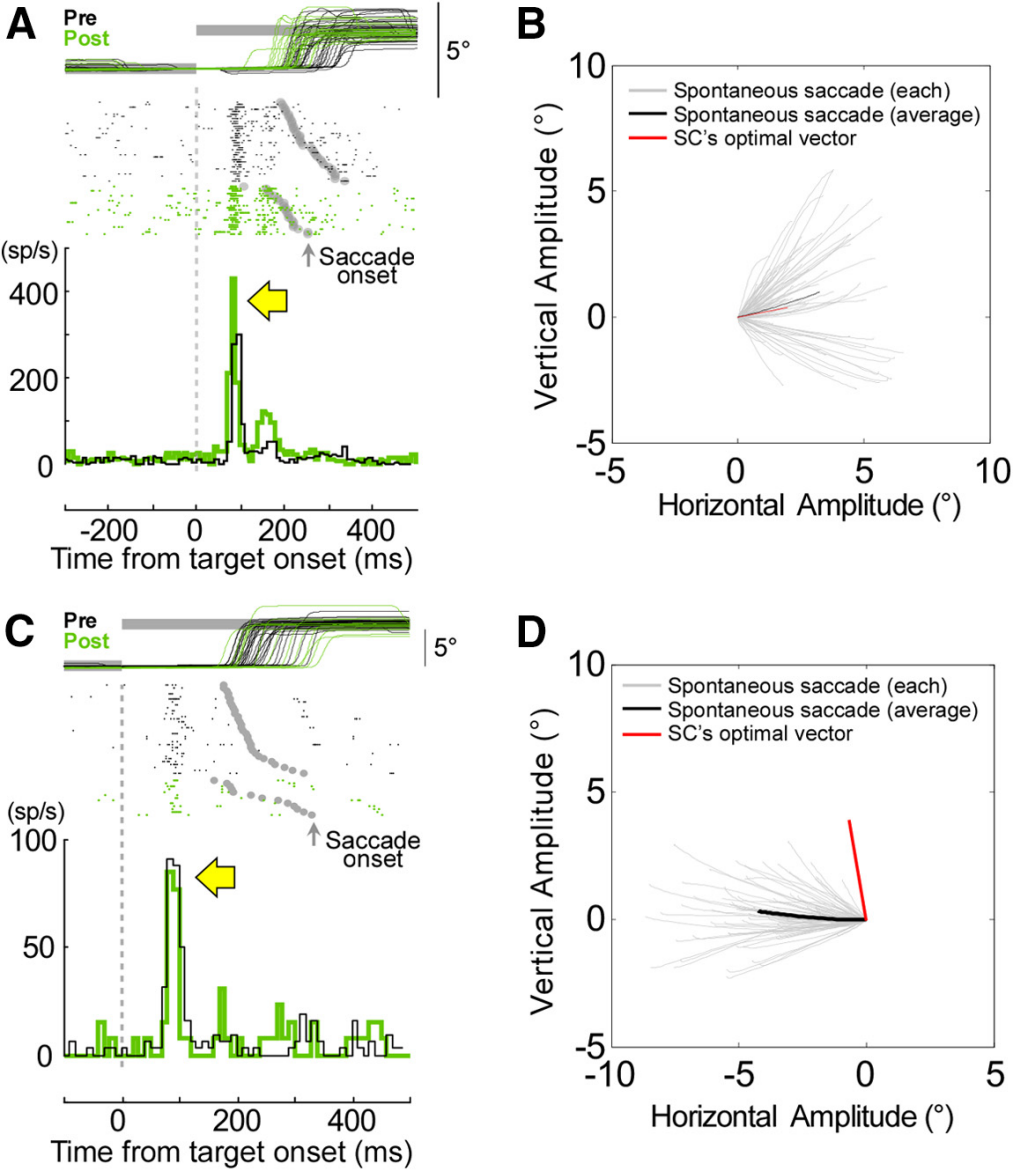
SC activity before and after SNr inactivation. ***A***, Visual response of a rostral SC neuron to a 2° target step inclined at 10° before (black) and after (green) SNr inactivation. The eye and target traces are horizontal components. Gray dots show times of saccade onsets. The visual burst associated with the target onset increased after inactivation (yellow arrow). ***B***, Trajectories of the spontaneous saccades caused by SNr inactivation (gray: first 75 individual saccades; black: average, 3° in amplitude inclined at 17°). Red line indicates the preferred vector of the SC neuron, i.e., 2° at 10°. ***C***, Visual response of another SC neuron to a 4° target step inclined at 100° before (black) and after (green) SNr inactivation. The eye and target traces are vertical components. The visual burst did not change after inactivation (yellow arrow). ***D***, Trajectory of the spontaneous saccades after SNr inactivation (gray: first 75 individual saccades; black: average (4° in amplitude inclined at 175°). Red line indicates the preferred vector of the recorded SC neuron (4° at 100°).

To determine the influence of SNr inactivation on SC error signal, we inactivated the SNr and compared the visual activity of SC neurons before and after the inactivation. Figure 9*A* compares the visual activity in the left rostral SC before (black) and after (green) left SNr inactivation (experiment #4). Consistent with a previous study (Hikosaka and Wurtz, 1985), the SNr injection did not affect the amplitude of contraversive saccades (Fig. 9*A*, top panel; pre: 1.99 ± 0.66°, post: 2.08 ± 0.79°, Wilcoxon rank-sum test *p* > 0.05). However, the injection did cause a significant decrease in their latency (pre: 248 ± 42 ms, post: 199 ± 34 ms, Wilcoxon rank-sum test *p* < 0.05). Importantly, the injection produced an increase in the cell’s visual activity [Fig. 9*A*, bottom panel, green (post) vs black (pre), yellow arrow]. After the injection, the average activity between 50 and 100 ms after the target step (vertical dotted line) was significantly greater (118 ± 35 spikes/s) than observed before the injection (92 ± 34 spikes/s; Wilcoxon rank-sum test *p* < 0.05).

Approximately 30 min after the injection, contraversive spontaneous saccades appeared (Fig. 9*B*, gray lines), which confirmed that the inactivation had been successful. The average vector of the spontaneous saccades (Fig. 9*B*, black line, 3° in amplitude inclined at 17°) was similar to the recorded SC neuron’s preferred vector (Fig. 9*B*, red line, 2° in amplitude inclined at 10°). Thus, in this experiment, the plotted vector of this neuron was within the range of the spontaneous saccades, suggesting that the inactivated SNr area is likely connected to the recorded SC neuron (Hikosaka and Wurtz, 1983b) and causes its increased visual activity.

Figure 9*C*,*D* shows data from another experiment (experiment #2), in which the plotted vector of the SC neuron was out of the range of the spontaneous saccades produced by SNr inactivation (Fig. 9*D*). Therefore, we presume that this inactivated SNr area did not project to the recorded SC neuron (Hikosaka and Wurtz, 1983b). In this experiment, SNr inactivation did not significantly change either the amplitude of the optimal saccade (pre: 5.60 ± 0.46°, post: 5.98 ± 1.18°, Wilcoxon rank-sum test *p* > 0.05; Fig. 9*C*, top panel) or its latency (pre: 217 ± 35°, post: 244 ± 59°, Wilcoxon rank-sum test *p* > 0.05). Appropriately, the unit’s visual activity did not increase after this ineffective SNr inactivation (Fig. 9*C*, bottom panel, yellow arrow). Comparison of the average activity seen 50–100 ms after the target step in trials before (40 ± 30 spikes/s) and after the injection (27 ± 22 spikes/s) showed no significant difference (Wilcoxon rank-sum test *p* > 0.05). Thus, SNr inactivation did not increase the visual activity if the SNr site did not project to the recorded SC neuron. These data support our hypothesis that the SNr modulates the SC visual sensitivity. Moreover, the modulation is vector specific.

## Discussion

This study demonstrates that inactivation of the SNr facilitates saccade adaptation, a type of error-based motor learning. After inactivation, the adaptation rate was significantly greater than normal in all of our datasets (Fig. 3). As reported previously (Hikosaka and Wurtz, 1985), muscimol inactivation of the SNr only modestly effects visually-guided saccades. In particular, the gain of the ipsiversive saccades that were to undergo adaptation in our experiments was unaffected in any of our inactivation experiments (Fig. 1*C*). Thus, our data suggest that the facilitated adaptation after SNr inactivation (Fig. 3) was not caused by muscimol directly modulating the action of SNr on ipsiversive saccades, but rather that it affected the adaptation process. Indeed, we saw changes in collicular neuron activity at the appropriate retinotopic location.

### Possible concerns about our conclusions

#### Muscimol could produce direct changes in saccade characteristics

Although most of the SNr projection to the SC is ipsilateral (∼85%), a small projection is contralateral (Jayaraman et al., 1977; Beckstead et al., 1981; Hikosaka and Wurtz, 1983b). Also, microstimulation of the SNr decreases the activity on both sides of the SC (Basso and Liu, 2007; Liu and Basso, 2008). Thus, SNr inactivation could possibly affect not only contralateral saccades, but ipsiversive saccades as well. However, we did not see evidence of such ipsilateral effects.

To establish that SNr inactivation affects adaptation, it is important that muscimol does not affect the initial ipsiversive saccade gain directly. In all 8 experiments (experiments #1–8), muscimol caused no significant change in the gain of 10° and 12° ipsiversive saccades (Fig. 1*C*). This finding is consistent with a previous study showing that SNr inactivation had no effect on 10° ipsiversive saccades (Hikosaka and Wurtz, 1985). However, the gain of ipsiversive saccades did decrease slightly after the injection (Figs. 1*C*, 4). To normalize this modest change across all experiments, we calculated the gain during adaptation relative to the median gain of the first 25 trials in each experiment. Also, the no-adaptation control experiments (Fig. 4) revealed that the slight gain change of ipsiversive saccades in injection experiments was not significantly different from no-injection experiments for the duration of the adaptation session. Thus, the faster gain change after SNr inactivation could not be attributed to a direct effect of muscimol on the metrics of ipsiversive saccades, and so must be an effect on the adaptation process.

#### Potential muscimol spread to other parts of the basal ganglia

Because the substantia nigra pars compacta (SNc) and subthalamic nucleus (STN) lie near the SNr, it is possible that the muscimol spread to those areas. However, the SNc and STN are located upstream of the SNr (Hikosaka et al., 2000), so any muscimol effects on these areas would not be transmitted through the inactivated SNr. Moreover, it is unlikely that the spread of muscimol to these areas affects the gain change because our control experiments showed no significant difference in the injection experiments (Fig. 4).

#### Gain change in no-injection control adaptations

As can be seen in Figure 3*C*, the gain changes in the no-injection control adaptation trial (colored bars) are smaller for experiments #1, 2, and 4 (Monkey E) than for experiments #5, 6, and 7 (Monkey Z). One possible explanation for this difference is that, for Monkey E, we started the postinjection adaptation paradigm (black bar) after the animal had already worked about one full hour (Table 1) because we used this monkey to collect the data presented in Figure 9. In contrast, for Monkey Z, we started adaptation only 30 min after it started working. The amount of adaptation in the control experiments for Monkey Z was comparable to that reported by others (Straube et al., 1997; Hopp and Fuchs, 2004; Kojima et al., 2015). Also, when we started adaptation 30 min after Monkey E started working, the amount of adaptation was comparable to that of the previous studies (Straube et al., 1997; Hopp and Fuchs, 2004; Kojima et al., 2015). We started the no-injection adaptation trials (for experiments #1, 2, 4, 5, 6, 7, 9) or no-adaptation trails (for experiments #3, 8) after the monkey had made about the same number of preinjection, during injection, and postinjection trials for the same amount of time as in an injection experiment. Thus, the greater gain change after SNr inactivation was not because of a small amount of no-injection adaptation.

#### Muscimol could affect visual and saccade related activity in the ipsilateral SC

Neurons in the rostral ipsilateral SC discharge not only for the visual error produced by a target step, but also burst for the corresponding saccade to the step (Kojima and Soetedjo, 2017b; Figs. 1*A*, brown arrow, 9). A previous study showed that the visual activity, which is induced by the constant visual error that drives saccade adaptation, decreased as the adaptation speed decreased. However, the bursts accompanying corrective saccade bursts did not (Kojima and Soetedjo, 2017b). This result suggests that the visual activity in the intermediate layer of the SC drives adaptation, not the corrective saccade bursts. Indeed, the timing of visual activity in the SC (Munoz and Wurtz, 1995; Sparks et al., 2000) and the enhancement of complex spike activity in the OMV during adaptation (Soetedjo and Fuchs, 2006; Soetedjo et al., 2008a,b) occur at about the same time, i.e., ∼80–100 ms after a target and adaptation step, respectively. Consequently, the corrective saccade-related burst is too late for the complex spikes to induce a plastic change in the OMV. Therefore, muscimol’s effect on this later saccade-related activity might not cause any effect on saccade adaptation. Additional experiments, such as recording of SNr activity during adaptation, could provide insight into a possible role of the visual and saccade-related activity of the SNr for saccade adaptation.

### A possible nigral influence on cerebellar plasticity

**Figure 10.**
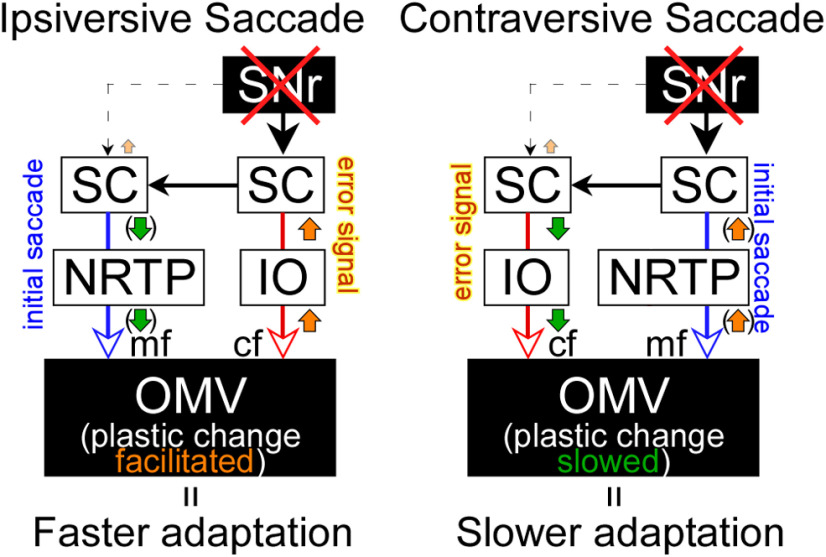
Schematic of connections from the SNr to the OMV underlying saccade adaptation effects. Red X marks indicate that the input from the SNr to the SC that is eliminated after a muscimol injection into the SNr. Orange and green arrows indicate the increase and decrease that occur in each structure after the SNr inactivation, respectively. Orange and green arrows in parentheses indicate the change does not affect the gain of initial saccade. SNr, substantia nigra pars reticulata; SC, superior colliculus; IO, inferior olive; NRTP, nucleus reticularis tegmenti pontis; OMV, oculomotor vermis; mf, mossy fibers; cf, climbing fibers.

How might SNr inactivation facilitate saccade adaptation? Since the SNr has a number of outputs, including nigrothalamic pathways that lead to the frontal eye fields, this cannot be absolutely specified. However, as shown in Figure 9, we did see changes in collicular activity that suggest a role for collicular output in this process. Figure 10 provides one possibility using a simplified block diagram to demonstrate the neural activity changes after right SNr inactivation and its effect on the plastic change in the cerebellum during gain decrease saccade adaptation. For ipsiversive saccade adaptation, the left SC produces a motor command for rightward initial saccades that reaches the oculomotor cerebellum (OMV; Fig. 10, blue pathway) via the nucleus reticularis tegmenti pontis (NRTP). During adaptation, the SC motor command signal for the initial saccade does not change or changes inconsistently (Frens and Van Opstal, 1997; Takeichi et al., 2007; Quessy et al., 2010), suggesting that the adaptation site of the plastic change is downstream of the SC.

Previous studies (Kaku et al., 2009; Soetedjo et al., 2009; Kojima and Soetedjo, 2017b, 2018) indicated that for adaptation of leftward visual error, the right SC’s visual sensory activity provides an error signal to the OMV via the IO (Fig. 10, red pathway) to drive the adaptation. As suggested by a cerebellar learning theory (Marr, 1969; Albus, 1971; Ito, 2005), this error signal increases complex spike activity, which induces plastic changes that reduce simple spike activity (Soetedjo and Fuchs, 2006; Soetedjo et al., 2008a,b; Kojima et al., 2010a) in the OMV. This decrease in simple spike activity decreases the saccade gain through downstream structures (Kojima, 2019; Soetedjo et al., 2019). In this study, we suppressed the SNr activity (red X sign). This increases the potency of the ipsilateral SC error signal (orange upward arrow) because the SNr no longer inhibits the ipsiversive SC (Fig. 9; Hikosaka et al., 2000). The increase of SC activity presumably increases the complex spike activity in the OMV (orange upward arrow) and facilitates the plastic changes in the OMV. These changes, in turn, facilitate the changes in the activity of downstream structures to speed up saccade adaptation.

In support of this suggestion, the SNr inactivation changed the kinematics of corrective saccades, which represent the error signal that drives saccade adaptation. Their reaction time was shorter and their peak velocity was higher in the injection experiments than in the no-injection experiments (Fig. 7*A*,*B*). Because the gain of corrective saccade amplitude did not change during adaptation (Fig. 7*C*), the changes in reaction time and peak velocity were not caused by amplitude changes in these saccades.

Interpreting the reaction time and peak velocity of the adapting saccades (Fig. 6) may not be straight forward because the amplitude changed during adaptation. The peak velocity of saccades in the injection experiments decreased more than the peak velocity of saccades in the no-injection experiments (Fig. 6*B*). This is consistent with the greater gain decrease observed in the injection experiments (Fig. 3). Because the peak velocity increased when gain did not change during the no-adaptation experiments (Fig. 6*D*), the decrease in peak velocity during adaptation was not brought about by fatigue induced by repetition of the task, but was instead because of the amplitude decrease. Saccade reaction time was longer in the injection experiments whether the amplitude changed or not (Fig. 6*A*,*C*). This extension of reaction time with little change in the peak velocity after inactivation is consistent with the findings of previous SNr inactivation studies (Hikosaka and Wurtz, 1985).

### A possible role for commissural connections

In addition to the direct SNr projection to the SC, commissural inhibition between the two sides of the SC needs to be considered (Olivier et al., 1998). The tectotectal projection includes both excitatory and inhibitory elements (Behan, 1985; Munoz and Istvan, 1998; Takahashi et al., 2010) and SNr is known to target both GABAergic and non-GABAergic neurons within the SC (Kaneda et al., 2008), but it is not known whether tectotectal neurons are targeted. Because commissural excitation is most likely used for vertical saccades (Takahashi et al., 2010), we will only consider commissural inhibition for the horizontal saccades that we used in this study. Thus, it is possible that inactivation of the SNr disinhibits the ipsilateral SC via the direct projection and then increases inhibition of the contralateral SC via the commissural projection (Fig. 10, green downward arrow). For ipsiversive saccades, the SNr inactivation would then simultaneously upregulate contralateral SC activity via the crossed nigrotectal projection and inhibit contralateral SC activity via the commissural pathway (small orange upward arrow). The increase in the reaction time of ipsiversive saccades (Fig. 6*C*) suggests the indirect effect via commissural inhibition is stronger than the direct effect of the crossed nigrotectal projection. Nevertheless, this increase is not enough to affect the initial saccade gain (Fig. 1*C*). For contraversive saccades, the activity of the ipsilateral SC is upregulated when ipsilateral SNr input is suppressed (orange upward arrow), while at the same time it is downregulated via indirect commissural inhibition (green downward arrow). The decrease in the reaction time of the contraversive saccade (Fig. 8*D*) suggests the direct effect of the ipsilateral nigrotectal projection is stronger than that of the commissural projection.

Commissural inhibition might also contribute to the slower adaptation of contraversive saccades (Fig. 8*B*). The gain of initial contraversive saccades in the injection datasets was not significantly different from the no-injection datasets (Fig. 8*C*), so it is possible to evaluate the effects of inactivation on the rate of adaptation in contraversive saccades. SNr inactivation could decrease the error signal in the contralateral SC via the commissural inhibition (Fig. 10, green downward arrow) leading to slower plastic changes in the OMV that in turn induce slower adaptation. Note that the combined effects of the SNr inactivation via direct projection and the commissural inhibition is weak with respect to saccades in either direction, since it has no significant influence on the gain. This weak influence might, nevertheless, be enough to affect the error signal that drives adaptation. Indeed, the adaptation can be induced by subthreshold SC stimulation (Kaku et al., 2009; Soetedjo et al., 2009) and the change in the SC activity for the error signal is only ∼10 spikes/s during adaptation (Kojima and Soetedjo, 2017b).

### A role for motivation in error-based motor learning

The effects of saccade adaptation seen in the present data are consistent with the results of previous studies. First, patients with Parkinson’s disease show slower saccade adaptation than age matched controls (MacAskill et al., 2002; Abouaf et al., 2012). The SNr is hyperactive in Parkinson’s disease patients (Wichmann et al., 1999; Wichmann and DeLong, 2003). Therefore, the slowing of adaptation in these patients is consistent with our finding that a hypoactive SNr produced by muscimol injections made adaptation faster. Second, our results also are consistent with reward motivation studies. Learning to use a robotic arm was facilitated by motivation in humans (Nikooyan and Ahmed, 2015), and saccade adaptation was facilitated by reward motivation in monkeys (Kojima and Soetedjo, 2017a). Motivation increases the activity of dopamine neurons, which leads, in turn, to decreases in SNr activity (Hikosaka et al., 2000; Yasuda et al., 2012). Note that in the previous behavioral study, which tested the motivation effects on saccade adaptation (Kojima and Soetedjo, 2017a), motivation affected the saccade motor command signal because the reaction time of adapting saccades shortened. The reaction time of corrective saccades, however, was not shortened. In contrast, the present inactivation study attempted to affect the error signal selectively (Fig. 1*A*), so the reaction times of the corrective saccades were shortened (Fig. 7), but those of the adapting saccades were not shortened (Fig. 6). Thus, it is possible that the neural mechanism of adaptations facilitated by the reward association and SNr inactivation could be different.

Artificially manipulating motivation level by reward association is one way to examine the motivational effects on the saccade adaptation. In this case, reward is associated with saccades in one direction and the adaptation effects are compared between rewarded saccades and unrewarded saccades (Kojima and Soetedjo, 2017a). There is another way to examine motivation effects during saccade adaptation. As the adaptation task is repeated, the adaptation speed decreases and reaction time increases, suggesting that motivation decays during adaptation. During this natural decay of motivation, the SC activity for the error signal is delayed and decreases (Kojima and Soetedjo, 2017b). The present study examined whether the SNr is involved in this error signal process, not the reward association for the saccade motor command signal, but together these two studies suggest the nigrotectal projection may produce motivation-related modification of collicular signals used for saccade adaptation. Nevertheless, this study does not provide direct evidence of whether motivation affects saccade adaptation through the SNr. To do so would require an examination of the inputs and internal processing within the SNr during saccade adaptation. Future studies are required to understand the mechanisms going on within each of the elements of the circuit.

Recently, an anatomic study showed the existence of subcortical pathways that interconnect the lateral cerebellum and the basal ganglia (Bostan and Strick, 2018). Ascending cerebellar projections from the dentate nucleus access the putamen by way of the intralaminar thalamus. It has been suggested that it is through this pathway that modifications in cerebellar activity produce dystonia, a disorder of the basal ganglia (Tewari et al., 2017). Descending basal ganglia projections from the STN access the lateral cerebellum by way of the pontine nuclei and the pedunculopontine nucleus (Bostan and Strick, 2018). This input potentially affects the simple spike activity of Purkinje cells through mossy fiber projections. Our study suggests a pathway from the SNr to the OMV via the SC. This input could potentially affect the complex spike activity of Purkinje cells. Thus, our results suggest a previously unrecognized function of the basal ganglia, in which it influences the error-based learning process in the cerebellum. Additional experiments demonstrating the presence of such a circuit, and the examining the activity within the components of the circuit during saccade adaptation will be required to test this hypothesis.

## Synthesis

Reviewing Editor: Mark Laubach, American University Decisions are customarily a result of the Reviewing Editor and the peer reviewers coming together and discussing their recommendations until a consensus is reached. When revisions are invited, a fact-based synthesis statement explaining their decision and outlining what is needed to prepare a revision will be listed below. The following reviewer(s) agreed to reveal their identity: Michael Goldberg.

Both reviewers felt that your manuscript would be improved dramatically and we all agreed that you should address their reviews in total. Looking forward to receiving your revised manuscript.

Reviewer #1

This an interesting paper that needs some work. The authors used muscimol inactivation of the substantia nigra pars reticulata to study the contribution of the SNr to saccadic adaptation. They took advantage of the previous observation that the superior colliculus provides, via the corrective saccade signal, the information that the cerebellum uses to drive the adaptation. They injected muscimol into a part of the SNr that evokes 4 degree (on the average) spontaneous saccades, and measured the process of gain-decreasing adaptation of contralateral saccades, which is driven by 4 degree ipsilateral corrective saccades. They found that the gain of contralateral saccadic adaptation to 4 degree ipsilateral target steps is increased by muscimol injection in to the SNr.

Unhappily, in their introduction, they do not actually describe this experimental design and motivation, nor in the discussion do they make it clear, that in gain decreasing ipsiversive saccades they induce four degree corrective saccades, precisely the saccades whose visual responses travel from the superior colliculus to the cerebellar vermis and induce the adaptation.

The authors write that for three experiments muscimol injected into the 4 degree region of SNr increased the gain of contraversive saccades, and therefore they did not look at saccadic adaptation in the contraversive direction. . They should show these data. It looks in Figure 1C that the gain of contraversive saccades is not affected by the intrasaccadic target step. Was this figure made from an experiment that had a gain change for contraversive saccades? Furthermore at 30 minutes both the ipsiversive and the contraversive saccades do not seem to be made to the original target without adaptation, which suggests that there was no gain increase for contraversive saccades. The authors should examine the adaptation in the cases in which the injection had no effect on the gain of the contraversive saccades to see if the adaptation took place.

eNeuro has no length limitation. The extended data, including the SC data should be in the main body of the paper.

Specific

Line 43 "no effect was seen on the amplitude of saccades when we did not induce adaptation" was this for ipsiversive saccades only?

Line 116-118. I assume that the authors found the region of the SNr not only associated with tonic activity during fixation and pauses during saccades, but they searched for areas associated with pauses associated with 4 degree and not 10 or 12 degree saccades. They should make this clear, unless I am wrong and they found the sweet spot by accident.

Figure 1B - the saccade is labeled ‘adapted saccade.’ The saccade is shown as landing on the target. If the saccade had been adapted, would not the saccade have been shorter than the target? The label should be ‘adapted saccade paradigm’ to make it clear that the saccade itself is not adapted in this example..

Figure 1C. The authors should label more adapted saccades with arrows, as well as labeling more corrective saccades, at all times. They should emphasize, perhaps in the legend, that the ‘spontaneous saccades’ were those induced by muscimol. It seems that the gain of spontaneous saccades were greater at 60 than at 30 minutes. Is this true? The authors write that the frequency of unadapted saccades increase with time, but they do not mention their amplitude. Furthermore the contraversive saccades do not seem to be adapted. Was this an example in which there was a gain increase for unadapted contraversive saccades?

Reviewer #2

The basal ganglia and cerebellum are involved in different types of motor learning, but recent studies suggest their interactions. This study demonstrated that inactivation of the substantia nigra pars reticulata (SNr) sped saccade adaptation. The authors trained two rhesus monkeys to track a small target spot with their eyes and induced adaptation by presenting an intrasaccadic backward target step. Previous studies showed that the changes in saccade amplitude during this adaptation paradigm depend on the cerebellum, and the superior colliculus (SC) transmits signals for both saccade generation and visual error that induce adaptation. During this task, the authors injected a small amount of muscimol into the SNr in an attempt to promote visual error signals in the ipsilateral rostral SC without altering the motor signals in the contralateral caudal SC. The authors found that the SNr inactivation facilitated gain-decrease adaptation of ipsiversive saccades, while the metrics of saccades remained unchanged in trials with no intrasaccadic target step. These findings are timely and uniquely contribute to the recent notions that computations in the cerebellum and the basal ganglia are interacted. However, I have several concerns about the interpretation of the results. In particular, the data show that inactivation altered several saccade parameters besides the time course of saccade adaptation, indicating that motor signals in the contralateral SC might be changed. I suggest a few major modifications that might be helpful for the authors to improve the manuscript.

1) Most of the analyses of saccade parameters were based on a linear mixed-effect model which estimated three coefficients, b1-3. It is reasonable that the authors evaluated the b3 coefficient (injection × trial interaction) when they examined the inactivation effects on the time course of saccade adaptation. However, the b1 and b2 values are also informative to understand the direct drug effects and the changes during adaptation. For example, in the most critical data in Figure 3, SNr inactivation seemed to reduce saccade gain throughout the block of non-adaptation trials. Therefore, I suggest the authors report all b1-3 values for Figs. 2-4 with appropriate estimates of effect size (for example, in %) and the results of statistical test (p-value) in an additional Table.

2) Muscimol spreads over time. Although the authors included only 472 saccade trials for their quantitative analysis, it is stated in the text of Extended Data that the post-injection adaptation experiments were started after monkey E had already worked one full hour. To clarify this point, please summarize the time of each adaptation experiment started (or ended) after injection in Table 1.

3) Latency of contraversive saccades decreased during the experiment shown in Extended Data Fig. 6. What about the other experiments? In relation to the point above, please add panels to Fig. 4 for contraversive primary saccades.

4) Significant changes in ipsiversive saccade latency (Fig. 4A) suggest that signals in the contralateral SC might be somehow altered during inactivation. This could be due to the direct crossed projection from the SNr to the contralateral SC, or to the commissural connection between both sides of the SC. If inactivation consistently reduced contraversive saccade latency, the balance between the colliculi might be altered, suggesting the latter possibility. As far as inactivation failed to alter saccade gain (as pointed in Major comment #1 above), the inactivation effects on saccade adaptation could be properly evaluated in the current setting. These points need to be discussed.

Minor

Please use larger fonts for figures, especially for the axis labels and statistical values.

Line 266: Fig. 6 may not have been properly quoted here.

Lines 424-427: These three sentences are not well connected.

Line 433: One paper from Fuchs lab (PMID: 17442764) showed changes in SC activity during adaptation.

The authors should carefully consider how to address this topic which came up in the discussion forum on the manuscript:

One reviewer wrote:

"...the authors should provide more friendly introduction to their experimental design and integrate all the extended data into the main text. A possible mechanism here might be muscimol spread to 10 deg site in the SNr and the balance of signals in both sides of the SC altered through the commissural connection. The authors can check this possibility by analyzing the data of contraversive saccades (latency and amplitude in non-adapted trials). Suppression in the contralateral SC (through facilitation in the ipsilateral SC) increased saccade latency (Fig. 4) but did not reduce amplitude (Fig. 3). We do not know about contraversive saccades. Even if that happens, the authors might be able to evaluate the inactivation effects on adaptation by evaluating the interaction (b3) component of their mixed-effect model, but they should explicitly state this important point."

The other reviewer replied:

"I do wonder about the contraversive saccades. In the experiments without SNr inhbition gain changes there should be the normal level of adaptation - the contraversive saccades should be adapted by ipsiversive corrective saccades, and these should be unaffected by the SNr inactivation."
